# Hip load capacity cut-points for Astronaut Skeletal Health NASA Finite Element Strength Task Group Recommendations

**DOI:** 10.1038/s41526-019-0066-3

**Published:** 2019-03-14

**Authors:** Andrew S. Michalski, Shreyasee Amin, Angela M. Cheung, Dianna D. Cody, Joyce H. Keyak, Thomas F. Lang, Daniel P. Nicolella, Eric S. Orwoll, Steven K. Boyd, Jean D. Sibonga

**Affiliations:** 1 Department of Radiology, Cumming School of Medicine, University of Calgary, Calgary, AB Canada; 2 McCaig Institute for Bone and Joint Health, University of Calgary, Calgary, AB Canada; 3 Biomedical Engineering Graduate Program, University of Calgary, Calgary, AB Canada; 4 Division of Epidemiology, Mayo Clinic, Rochester, MN USA; 5 Division of Rheumatology, Department of Internal Medicine, Mayo Clinic, Rochester, MN USA; 6 Department of Physics, Ryerson University, Toronto, ON Canada; 7 Centre of Excellence in Skeletal Health Assessment, Joint Department of Medical Imaging, University of Toronto, Toronto, ON Canada; 8 Osteoporosis Program, University Health Network, Toronto, ON Canada; 9 Department of Imaging Physics, University of Texas MD Anderson Cancer Center, Houston, TX USA; 10 Department of Radiological Sciences, University of California, Irvine, CA USA; 11 Department of Mechanical and Aerospace Engineering, University of California, Irvine, CA USA; 12 Department of Biomedical Engineering, University of California, Irvine, CA USA; 13 Department of Radiology and Biomedical Imaging, University of California, San Francisco, San Francisco, CA USA; 14 Musculoskeletal Biomechanics Section, Materials Engineering Department, Southwest Research Institute, San Antonio, TX USA; 15 Bone and Mineral Unit, Oregon Health Sciences University, Portland, OR USA; 16 Department of Radiology, Cumming School of Medicine, University of Calgary, Calgary, AB Canada; 17 McCaig Institute for Bone and Joint Health, University of Calgary, Calgary, AB Canada; 18 Division of Biomedical Research and Environmental Sciences, NASA Lyndon B. Johnson Space Center, Houston, TX USA

## Abstract

Concerns raised at a 2010 Bone Summit held for National Aeronautics and Space Administration Johnson Space Center led experts in finite element (FE) modeling for hip fracture prediction to propose including hip load capacity in the standards for astronaut skeletal health. The current standards for bone are based upon areal bone mineral density (aBMD) measurements by dual X-ray absorptiometry (DXA) and an adaptation of aBMD cut-points for fragility fractures. Task Group members recommended (i) a minimum permissible outcome limit (POL) for post-mission hip bone load capacity, (ii) use of FE hip load capacity to further screen applicants to astronaut corps, (iii) a minimum pre-flight standard for a second long-duration mission, and (iv) a method for assessing which post-mission physical activities might increase an astronaut’s risk for fracture after return. QCT-FE models of eight astronaut were analyzed using nonlinear single-limb stance (NLS) and posterolateral fall (NLF) loading configurations. QCT data from the Age Gene/Environment Susceptibility (AGES) Reykjavik cohort and the Rochester Epidemiology Project were analyzed using identical modeling procedures. The 75^th^ percentile of NLS hip load capacity for fractured elderly males of the AGES cohort (9537N) was selected as a post-mission POL. The NLF model, in combination with a Probabilistic Risk Assessment tool, was used to assess the likelihood of exceeding the hip load capacity during post-flight activities. There was no recommendation to replace the current DXA-based standards. However, FE estimation of hip load capacity appeared more meaningful for younger, physically active astronauts and was recommended to supplement aBMD cut-points.

## Introduction

Skeletal changes with spaceflight have been known to exist since early missions;^1,2^ however, a clinical risk for fracture or for osteoporosis as a consequence of microgravity-induced bone loss is not readily seen. Since the completion of the International Space Station (ISS) in 2000 and with the increased duration of future spaceflights, some of which are planned beyond low Earth orbit, National Aeronautics and Space Administration (NASA) has been concerned with microgravity-induced bone loss^3–6^ and its effects on skeletal integrity after the return from flight.^7–9^ Spaceflight is known to induce declines in hip load capacity,^10^ which may increase the factor of risk for hip fracture (where applied mechanical loads> bone load capacity). Therefore, it is plausible that a fracture could occur during the performance of activities (either during or after flight) that would otherwise be unlikely before spaceflight.

**Fig. 1:**
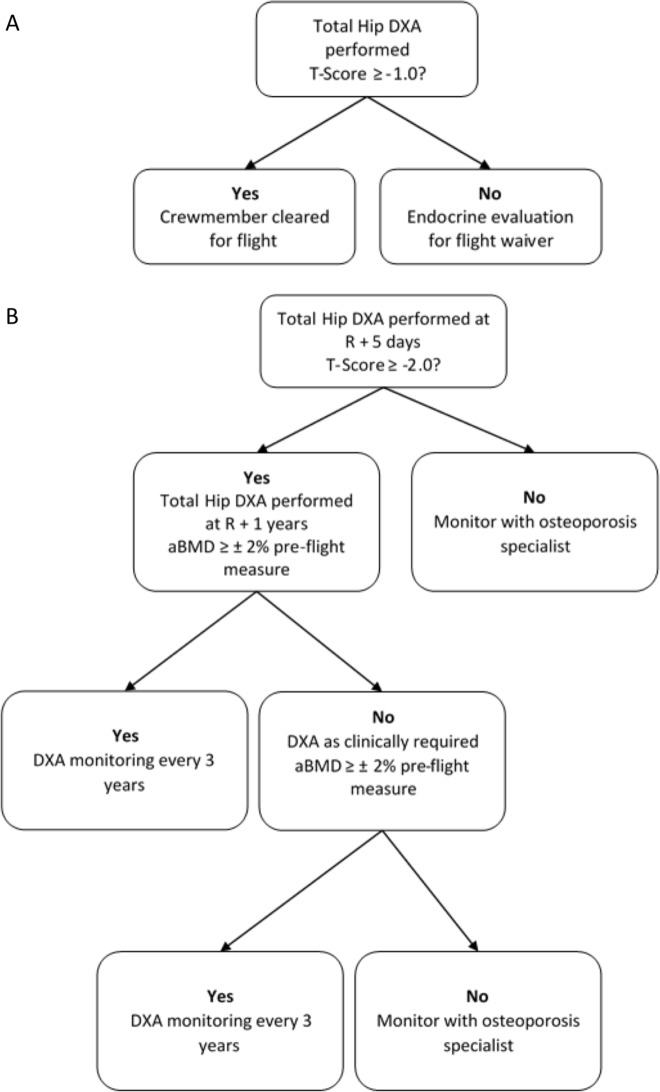
**a** Current pre-flight standard operating procedure for skeletal assessment. **b** Current post-flight standard operating procedure for skeletal assessment. R refers to time point for return from mission

Astronaut bone health medical standards are established to set operating bands (acceptable ranges) of bone health measurements in the long-duration astronauts. These operating bands of astronaut skeletal health are an adaptation of guidelines formulated by the World Health Organization (WHO) to diagnose primary osteoporosis, a condition of skeletal fragility due to aging that predisposes humans to low trauma fractures. The WHO guidelines are based upon measurements of areal bone mineral density (aBMD) at the hip by dual X-ray absorptiometry (DXA). Spine skeletal health is also of concern, but modifications to those standards are beyond the scope of this investigation. A pathway for using pre- and post-flight DXA testing to drive the protection of astronaut skeletal health is outlined in Fig. 1a, b, respectively. Based on current medical standards, an astronaut is certified for long-duration spaceflight by a pre-flight hip aBMD measurement of 1 SD below the mean young sex-matched population reference aBMD (i.e., *T*-score ≥ − 1.0) or greater. An astronaut’s post-flight acceptable aBMD cut-point (the “permissible outcome limit” = POL) is a *T*-score ≥ − 2.0. Hence, effective countermeasures are aimed at maintaining aBMD consistent with the POL and post-flight rehabilitation is targeted to restore aBMD to baseline levels (within the measurement error of the DXA test).^11^

As the WHO guidelines were originally developed from and for a population with age-related bone loss, NASA convened experts in osteoporosis and bone densitometry in Houston, TX, USA, for a NASA Bone Summit. A goal for the Bone Summit was to solicit opinions on the assessment of skeletal health in the younger-aged astronauts before and after spaceflight. After an in-depth review of biomedical and research data from astronauts exposed to long-duration spaceflight, the members of the Bone Summit highlighted the inability of DXA measurements to capture the full effects of spaceflight^12^ and expressed concern that the sole measurement of aBMD was insufficient for understanding space-induced changes to skeletal health, particularly for this understudied (younger, active, predominantly male) population exposed to a unique skeletal insult. Notably, DXA has multiple limitations (e.g., two-dimensional measurement, poor geometric representation, and lack of compartmental and micro-architecture characterization).^13–16^ Consequently, to maintain the astronaut’s high standard of skeletal health, the current clinical test (DXA) for osteoporosis needs to be expanded and state-of-the-art technologies may need to be used, to provide a comprehensive detection of spaceflight-induced changes to the hip and a greater understanding of how those changes influence hip bone load capacity. The experts suggested that NASA investigate using estimates of hip bone load capacity—from the analysis of quantitative computed tomography (QCT)-derived finite element (FE) models—to interpret and assess the skeletal health status of current astronauts and potential astronaut candidates.

FE models incorporate multiple determinants of bone load capacity, including density, geometry, and size. In addition, prospective studies have documented that both QCT and FE modeling can independently assess hip fracture risk in comparison with DXA aBMD alone.^17–22^ Without incorporating additional QCT assessment of skeletal health, NASA is at risk of inadequately observing skeletal changes with spaceflight and recovery, poorly estimating countermeasure efficacy, and not identifying at-risk crewmembers that may require further skeletal protection.^23,24^ Following the review of both astronauts’ clinical and biomedical research data, summit members noted that DXA technology for BMD measurements did not detect all the skeletal changes, as detected by QCT. Even though there are no clinical guidelines associated with QCT measurements, the data acquired describe profound skeletal changes that should be monitored as the utility of those measurements are being defined.^12^ Consequently, it was perceived that estimations of hip bone load capacity by QCT-FE modeling could help describe spaceflight effects on a function (i.e., the hip resistance to fracture under specifically oriented applied loading).^12^ Moreover, there is ongoing application of QCT-FE modeling to terrestrial population studies^20,21,25,26^ to establish clinical guidelines. Consequently, the Bone Discipline in the NASA Human Research Program explored how FE modeling could be used to set skeletal health standards for astronauts.

To this aim, the FE Strength Task Group I (2011) was convened, which was composed of investigators who have applied QCT-FE modeling in terrestrial studies of aging populations. The FE Strength Task Group I recommended that a dataset be developed of QCT-FE hip load capacity estimates from subjects whose ages span the range of active astronauts (35–55 years). For these specific purposes, QCT hip scans from population studies were donated by members of this Task Group I, who either served as or acted on behalf of the principal investigators of the studies. A single FE modeling approach was applied to all hip QCT scans to ensure consistency in hip bone load capacity estimations. In 2016, FE Strength Task Group II was convened to review FE data from combined population studies^27–29^ and to recommend the hip load capacity cut-points to establish operating bands of astronaut health. These proposed FE load capacity cut-points would complement the DXA aBMD-based *T*-scores currently used as the NASA standard for astronaut skeletal health.

In this report, the FE Strength Task Group II recommends use of QCT-FE cut-points to establish (i) a minimum FE estimate of hip load capacity after a mission as a POL, (ii) an additional skeletal health index to qualify an applicant for astronaut candidacy for further medical screening, (iii) a minimum fitness-for-flight standard for second long-duration mission, and (iv) an index by which flight surgeons could assess whether certain post-mission physical activities might increase the astronaut’s risk factor for fracture. Application of these FE-based standards of bone health with flight data from astronauts (*n* = 8) are demonstrated.

## Results

### Population-derived FE load capacity cut-points

**Fig. 2:**
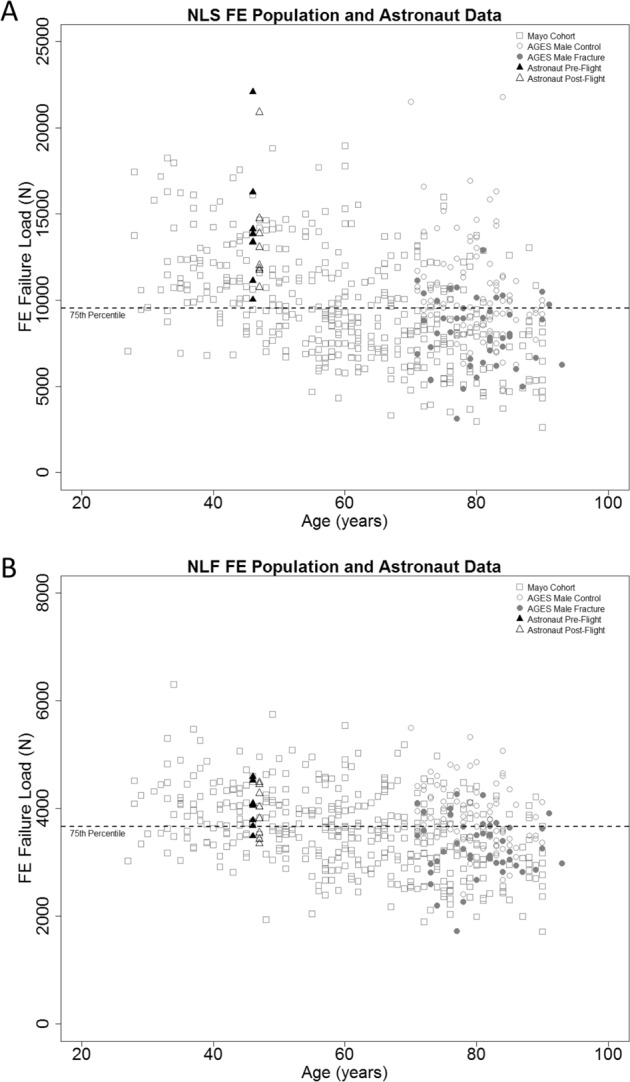
**a** Nonlinear stance subject data with age and FE load capacity, the dashed line represents the 75^th^ percentile FE load capacity cut-point at 9537N. **b** Nonlinear fall population data with age and FE load capacity, the dashed line represents the 75^th^ percentile FE load capacity cut-point at 3664N. The 75^th^ percentile is defined from the AGES male fracture cohort. Astronaut data are represented at a single time point to maintain non-identifiable data

**Fig. 3:**
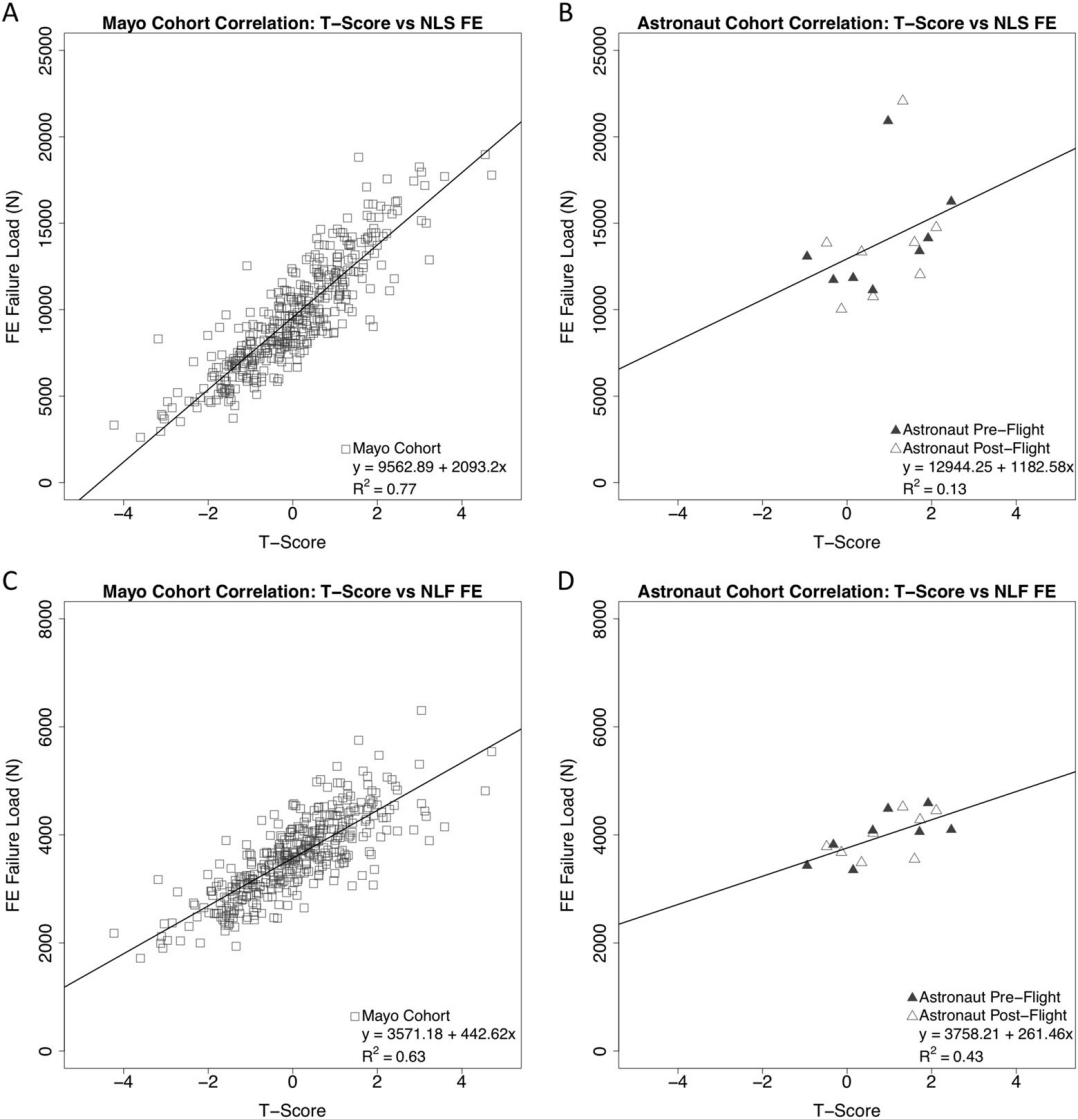
**a** Correlation data for the Mayo cohort between DXA *T*-score and NLS FE outcomes. *R*^2^ = 0.77. **b** Correlation data for the astronaut cohort between DXA *T*-score and NLS FE outcomes. Pre- and post-flight data are analyzed as a combined set. *R*^2^ = 0.13. **c** Correlation data for the Mayo cohort between DXA *T*-score and NLF FE outcomes. *R*^2^ = 0.63. **d** Correlation data for the astronaut cohort between DXA *T*-score and NLF FE outcomes. Pre- and post-flight data are analyzed as a combined set. *R*^2^ = 0.43

Demographic data for all cohorts used in the data analysis can be referenced in Table 1. FE analysis was implemented using nonlinear material properties for the models.^29^ Population data from the nonlinear single-limb stance (NLS) and nonlinear posterolateral fall (NLF) FE analyses are presented in Fig. 2a, b, respectively. All astronaut data are represented at a single age point and flight duration to ensure data are non-identifiable. As per Task Group assessments, a 75^th^ percentile cut-point, as determined from the Age Gene/Environment Susceptibility (AGES) Reykjavik male fractured cohort, was selected as the POL. The 75^th^ percentile for NLF FE load capacity was 3664N and the NLS FE load capacity 9537N. The 75^th^ percentile, vs. the mean or median load capacity of the AGES cohort, sets a higher standard for countermeasure efficacy and for skeletal protection in the younger-aged astronaut with many lifetime years remaining. For the stance loading configuration, the pre-flight and post-flight data of astronauts (*n* = 8 tested) both exceeded the FE load capacity POL. For the fall loading configuration, the hip load capacity of one astronaut falls below this POL cut-point before spaceflight and hip load capacities for three astronauts fall below the POL cut-point after spaceflight. Data included show, on average, that astronaut pre-flight (NLS: 14279 ± 3673N) and post-flight (NLS: 13624 ± 3215N) hip load capacities are significantly greater (*p* < 0.05) than the population mean (NLS: 11416 ± 2816N) for a subset of the Mayo cohort (ages 27–55 years). Figure 3 depicts correlations between the DXA *T*-Scores and FE outcomes for the Mayo cohort and the astronaut cohorts (combined pre- and post-flight outcomes). The population DXA data are moderately correlated and statistically significant with the FE estimated NLS (*R*^2^ = 0.77, *p* < 0.001) and NLF (*R*^2^ = 0.63, *p* < 0.001) hip load capacities. The astronaut DXA data are poorly correlated with the NLS (*R*^2^ = 0.13, *p* > 0.05) and NLF (*R*^2^ = 0.43, *p* < 0.01) load capacities, where the NLS correlation is not statistically significant (Table 2).

**Table 1:** Cohort information for the AGES, Mayo, and astronaut cohorts

AGES cohort	Non-fractured cases	Fracture cases
*N*	94	45
Age (years)	79.6 ± 5.2 (70.0–90.0)	80.3 ± 5.7 (71.0–93.0)
Height (cm)	174.9 ± 6.7 (162.4–190.5)	175.1 ± 5.5 (159.2–187.1)
Body weight (kg)	82.6 ± 14.8 (52.4–135.0)	80.1 ± 14.4 (50.3–114.0)
**Mayo cohort**	**Males**	**Females**
*N*	181	216
Age (years)	59.9 ± 16.6 (28.0–90.0)	60.9 ± 14.5 (27.0–90.0)
Height (cm)	176.3 ± 7.2 (157.0–195.4)	162.3 ± 6.1 (148.5–179.2)
Body weight (kg)	90.2 ± 16.5 (53.0–134.0)	74.4 ± 15.4 (51.0–128.5)
**Astronauts**		
*N*	8 (Males = 6, females = 2)	
Age (years)	46.0 ± 5.0 (38.0–53.0)	
Height (cm)	176.0 ± 5.0 (168.0–180.0)	
Body weight (kg)	72.0 ± 12.0 (59.0–88.0)	
Duration of flight (days)	154.0 ± 19.0 (125.0–169.0)	

All values presented as mean ± SD (range)

**Table 2:** DXA and QCT-FE results for the astronaut cohort

Astronaut cohort	Pre-flight	Post-flight	
DXA scan time (days)	95 ± 34 (56–160)	8 ± 2 (5–11)	
Total hip aBMD (g/cm^2^)	1.06 ± 0.13 (0.88–1.24)	1.03 ± 0.13 (0.83–1.20)	
Total hip *T*-score	0.97 ± 1.04 (−0.48–2.46)	0.74 ± 1.07 (−0.95–2.11)	
QCT scan time (days)	82 ± 26 (42–123)	10 ± 2 (6–14)	
*F*_NLS_ (*N*)	14279 ± 3673 (10,030–22,072)	13624 ± 3215 (10,757–20,914)	
*F*_NLF_ (*N*)	4037 ± 385 (3488–4590)	3925 ± 456 (3350–4486)	
Total hip aBMD	−0.03 ± 0.02	Total hip aBMD	−2.73 ± 1.93
Absolute change (g/cm^2^)	(−0.06–0.001)	Relative change (%)	(−6.39–0.10)
Total hip *T*-score	−0.23 ± 0.15	Total hip *T*-score	15.28 ± 67.12
Absolute change	(−0.46–0.01)	Relative change (%)	(−57.94–140.83)
*F*_NLS_	−654.25 ± 1239.04	*F*_NLS_	−3.63 ± 6.93
Absolute change (*N*)	(−2106–1697)	Relative change (%)	(−14.90–16.92)
*F*_NLF_	−111.50 ± 279.32	*F*_NLF_	−2.74 ± 6.93
Absolute change (*N*)	(−507–354)	Relative change (%)	(−12.49–8.64)

All values presented as mean ± SD (range). *F*_NLS_ and *F*_NLF_ refer to load capacities for the nonlinear stance and nonlinear fall loading configurations, respectively. Absolute and relative changes are reported as change from pre-flight values

### An assessment tool for bone health

**Fig. 4:**
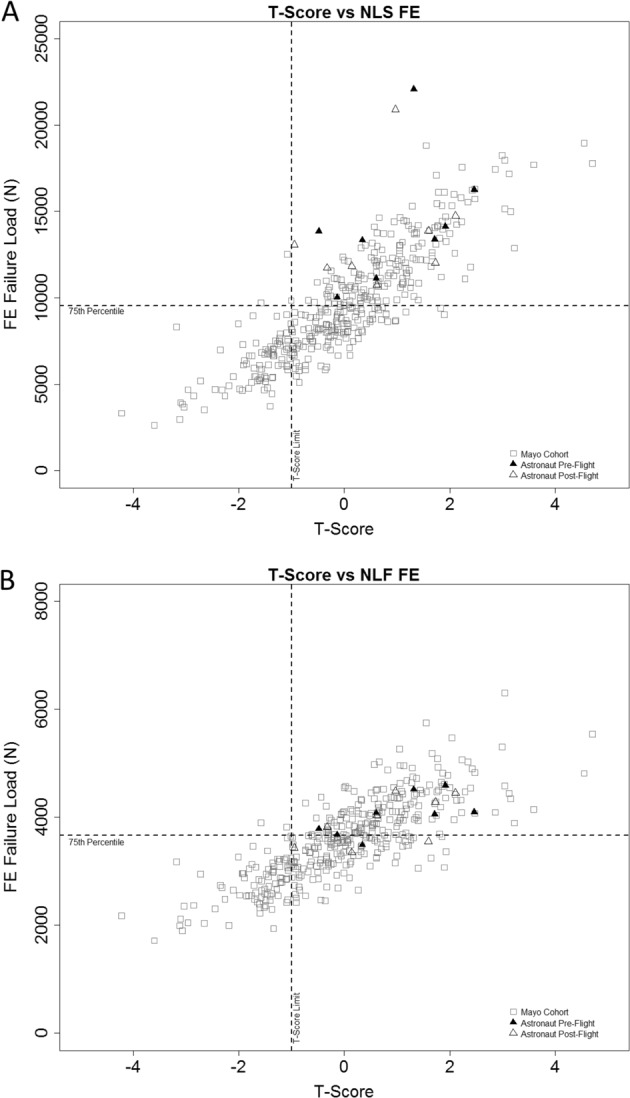
**a** Nonlinear stance clinical chart with *T*-score and FE load capacity. The vertical dashed line represents the DXA clinical standard at a *T*-score of −1.0 and the horizontal dashed line represents the 75^th^ percentile FE load capacity cut-point at 9537N. **b** Nonlinear fall clinical chart with *T*-score and FE load capacity. The vertical dashed line represents the DXA clinical standard at a *T*-score of −1.0 and the horizontal dashed line represents the 75^th^ percentile FE load capacity cut-point at 3664N

Figure 4a, b illustrate how the combined cut-points would differentiate skeletal health in the younger-aged terrestrial Mayo population and the astronaut cohort. Cut-points include the proposed FE-derived hip load capacity (NLS = 9537N and NLF = 3664N) relative to the DXA *T*-score of −1.0. These charts propose an additional metric to DXA *T*-score for screening bone health in applicants to the astronaut corps who fail to meet the bone medical standard of hip *T*-score > −1.0.

## Discussion

### Review of current astronaut skeletal health standards

In terrestrial medicine, the probability of fracture in the population at risk for age-related bone loss^11,30,31^ undergirds the WHO criteria for interventions at BMD *T*-scores < −2.5 for the hip, spine, or wrist.^32^ The WHO DXA *T*-score guidelines have minimal value for applicants for astronaut candidacy and for many active astronauts because of their age-range (35–55 years). The WHO DXA guidelines were never intended to assess a population experiencing bone loss due to prolonged spaceflight. To date,^11^ all astronauts have returned from missions on the ISS with BMDs above the POL (*T*-score = −2.0 SD). However, the use of *T*-scores alone may inadequately characterize any risks associated with rapid and significant BMD losses (10–15%^15^) observed after spaceflight. Furthermore, these data suggest that the POL standard is not sensitive for monitoring the efficacy of in-flight countermeasures since astronauts were meeting the standard before mitigation of bone loss by on-orbit use of bisphosphonates or the Advanced Resistive Exercise Device (ARED) was observed.^33,34^

As previously identified at the Bone Summit, DXA fails to detect changes to cortical and trabecular sub-regions of the hip.^12^ DXA is limited by the two-dimensional nature of the imaging modality, which lacks the ability to accurately characterize bone size and geometry. DXA’s also averages total bone mass^35^ across bone compartments, where the highly dense mineral in cortical obscure any changes to trabecular bone mass. In terrestrial studies, these limitations contribute to the poor predictability of fragility fractures in both men and women, who have not reached the diagnostic BMD cut-point for osteoporosis.^36^ Unlike elderly subjects, young individuals, such as the astronaut cohort, are more likely to fracture due to excessively overloading their bones caused by sports or other trauma,^37,38^ rather than osteoporosis, which reduces the utility of WHO guidelines^32^ for the astronaut cohort.

Following the review of astronaut biomedical data, both research and clinical, Bone Summit experts additionally recommended that an index of skeletal integrity (e.g., bone load capacity) could be serially evaluated in long-duration astronauts and may enhance the evaluation of astronaut eligibility. The effects of microgravity on bone integrity are not well understood and require more thorough evaluation. However, in a position development by the International Society of Clinical Densitometry (ISCD), QCT-FE has been identified as a rigorous and repeatable approach to obtain a composite assessment of bone integrity^39^ and has been recommended by the ISCD for use to monitor changes in skeletal health related to age or treatment,^40,41^ in addition to DXA. This study suggests that, because FE estimates of hip load capacity integrates biomechanics in its evaluation, it likely has utility for assessing skeletal health in the physically active astronaut, after spaceflight.

The standard development of clinical practice guidelines for bone health in terrestrial medicine considers a multitude of data from large population studies with fracture outcomes. The astronauts, in contrast, represent an understudied target population for bone loss (physically fit, healthy, predominantly male, exposed to spaceflight) for which there are limited baseline data. Group mean data from astronauts can also be confounded by large variations in subject-specific characteristics, including age, training, flight duration, and flight conditions. Although DXA offers an epidemiological approach using BMD *T*-scores to convey a relative risk for fracture, these *T*-scores do not identify who will fracture. The FE bone load capacity may be a more meaningful measure for evaluating the astronaut after spaceflight, because QCT-FE facilitates an individualized biomechanical risk assessment of hip fractures that is specific to the astronaut’s geometry, bone density distribution, bone size, and shape of the hip. As exhibited in Fig. 3b, astronauts may have similar hip load capacities but highly variable DXA *T*-scores. This observation is even more apparent in the Mayo cohort data (Fig. 3a), suggesting that the variability from using DXA is greater than the variability and precision expected of QCT-FE. By incorporating this new index for skeletal health that integrates more skeletal attributes influenced by spaceflight, a customized risk management plan can be developed specific to each astronaut. There is a potential for enhancing the assessment of fracture risk probabilities and the management of spaceflight-induced bone loss.

### Recommendation 1

The FE Strength Task Group recommends using QCT-FE data from population studies to assess overall skeletal health of the hip in astronauts. Based upon the analysis of population fracture data, a POL of 9537N for the hip in the NLS loading configuration is recommended. This FE load capacity cut-point should be used as an additional pre- and post-flight skeletal health index to evaluate astronauts. Furthermore, this FE load capacity cut-point is applicable for both male and female astronauts. The same POL should also be used as a minimum standard to measure countermeasure efficacy for astronauts.

A previous study used the WHO *T*-score classifications to determine FE interventional cut-points—for a sideways fall hip loading configuration^22^—which were equivalent to osteoporotic BMD classification (3000N for women and 3500N for men), thereby allowing for a clinical interpretation of FE hip load capacity. However, just as with DXA *T*-scores in young humans,^32^ intervention cut-points are driven by fracture probability and fractures in the elderly population are not equivalent to fractures in young, healthy persons.^37^ In addition, the FE Strength Task Group II recommends using a single cut-point for both male and female astronauts, not only for ease of implementation but to hold NASA to a higher standard for protecting skeletal health. On average, women have lower FE estimated hip load capacity, presumably due to menopause-induced bone loss and smaller bones, leading to an inherent fracture risk at lower applied loads to hip. Based upon observations of the Mayo cohort in Fig. 2, it is not unreasonable to hold female astronauts to this criterion, as many young females in this terrestrial cohort exceed the 75^th^ percentile cut-point.

Previous spaceflight work shows that DXA may not capture all the changes that occur in bones during spaceflight. Lang et al.^15^ showed higher rates of bone loss in the trabecular compartment, as measured by QCT, compared with the DXA rates of total bone loss in the hip. Furthermore, using QCT, Carpenter et al.^42^ demonstrated that vertebral trabecular volumetric bone mineral density (vBMD) remained lower than pre-flight values after 2–4 years of recovery, whereas DXA vertebral aBMD showed a recovery in bone density for the identical astronauts. A recent study has shown that tibial cortical porosity and trabecular BMD, as measured by high-resolution peripheral QCT, fail to recover after a year from returning from spaceflight.^43^ Spaceflight-induced bone loss persisting after return to Earth could combine with age-related bone loss and prematurely increase the fracture risk for astronauts later in life.

The FE Strength Task Group recommends that the FE load capacity POL be used as an additional standard for countermeasure efficacy (e.g., ARED exercise prescriptions). In terrestrial populations, the ISCD highlights the use of QCT-FE^40,41^ to monitor changes in bone health and load capacity. Terrestrial studies also show that exercise induces changes in bone size,^44^ which are detected by QCT and not DXA; these changes are incorporated into QCT-FE models, suggesting greater sensitivity for detecting the effects of exercise.

### Recommendation 2

The NASA Probabilistic Risk Assessment (PRA) model for fracture probabilities can be used to assess fracture likelihoods for both mission-specific tasks and post-mission activities. A model of linear bone deconditioning may be convenient for assessing fracture probabilities with varying durations of spaceflight exposures but efforts to develop a physiologically relevant model for skeletal deconditioning over time are warranted for this rare and unique skeletal insult. The combination of FE models to estimate hip load capacity and the PRA model to assess the range of physical activities that might mechanically overload the hip could have clinical utility for managing fracture risk in physically active persons.

Risk assessment in terrestrial populations is performed using specific risk assessment tools (e.g., FRAX), which integrate multiple determinants and risk factors for fracture, including BMD, to assess fracture probability and eligibility for treatment.^45^ However, these fracture risk assessment tools fail to integrate bone loss due to spaceflight or prolonged disuse and are not grounded in data from younger individuals. There is currently no fracture risk assessment tool that incorporates QCT-FE outcomes. Nevertheless, QCT-FE has been used for fracture prediction in terrestrial cohorts. The nonlinear stance analysis used in this work has improved fracture prediction (NLS *R*^2^ = 0.82, NLF *R*^2^ = 0.53) for both men and women, compared with the nonlinear fall loading configuration.^29^ Therefore, the FE Strength Task Group II recommends the use of the NLS loading configuration for a skeletal health standard to monitor hip biomechanical integrity in astronauts. In contrast, the NLF loading configuration is a conditional loading configuration, which requires a fall to the hip for the analysis to be relevant; even if a fall occurs, the impact force of may differ from the NLF loading condition.

**Fig. 5:**
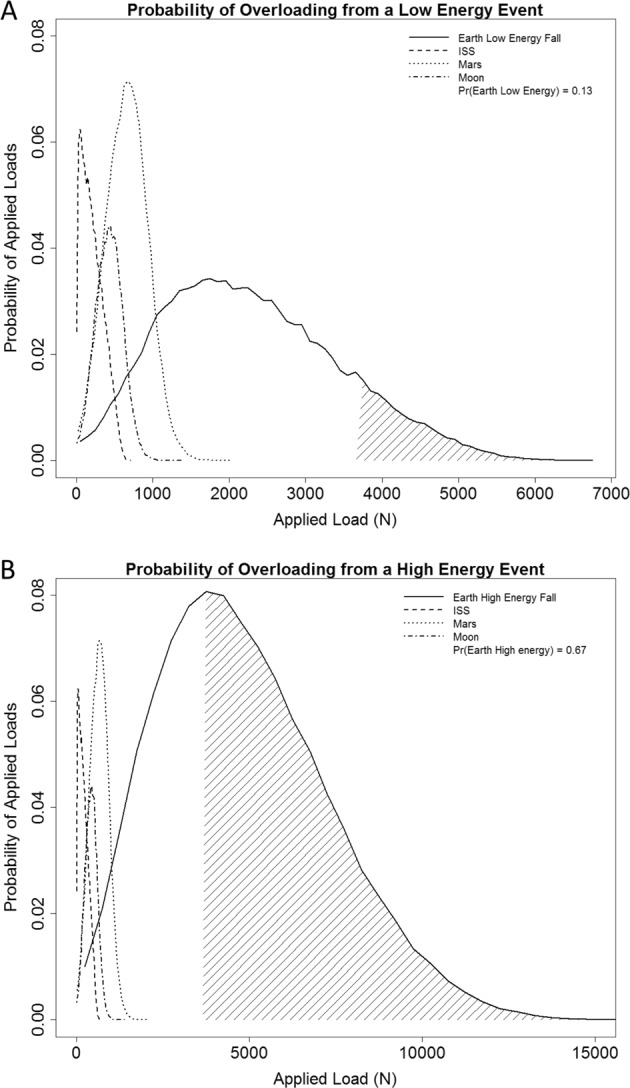
Application of the PRA model to determine fracture risk. Each line reflects the distribution of loads for a specific mission with a different exposure to gravity (e.g., ISS, Moon, Mars, and return to Earth). Probabilities are based on an assessed NLF bone load capacity equivalent to the NLF cut-point of 3664N. **a** The distribution of loads expected to be applied to the hip for different missions for low-energy events (loads between 1–4 kN). The probability of experiencing an overloading event after return to Earth is 0.13 (e.g., tripping and falling). **b** Assessment of probability of fracture using the NLF load capacity cut-point after a long-duration spaceflight mission and return to Earth for high-energy events (e.g., collision while playing football). The probability of experiencing an overloading event is 0.67

NASA previously determined a standardized PRA methodology, to establish mission-specific fracture risk from loading scenarios, including a fall on the hip.^46^ PRA uses a biomechanical model of a sideways fall on the hip to estimate a probability distribution of an overloading fracture. The biomechanical model incorporates multiple parameters, including gravitational forces and mission-specific parameters that contribute to skeletal loading.^46^ Combined with the NLF FE data, PRA incorporates a distribution of applied loads at the hip (Fig. 5a) for mission-specific tasks or post-mission activities, to determine the hazard risk for fracture with low- and high-energy falls. Based on Monte Carlo simulation, load distributions for missions to the ISS, Moon, and Mars have been included to compare with loads after return to Earth. Fracture risk in younger persons is mainly due to excessive force on the bone,^37^ which would be the primary concern for astronauts prematurely resuming activities with the potential for high forces (e.g., playing contact football, skydiving). For low-force events (Fig. 5a), there is a probability of 0.13 for bone overload using the NLF load capacity cut-point. Using PRA and the NLF FE load capacity cut-point (Fig. 5b), there is a probability of 0.67 for bone overload for high-force events using the NLF load capacity cut-point. The large reported probabilities for overloading are due to the high loading levels associated with the high-energy activities. The FE Strength Task Group II recommends using the NLF FE load capacity of individual astronauts to assess probabilities of hip fractures during the performance of in-mission tasks and of post-mission activities.^46^ Based on load distributions for various missions, there is little to no risk of experiencing an overloading fracture during the microgravity missions to the ISS, the Moon, or Mars. However, these probabilities do not account for any associated physiological deconditioning with long-duration spaceflight (e.g., muscle atrophy), which can increase the risk for falls.

### Recommendation 3

There are no new recommendations for qualifying applicants for astronaut candidacy or for certifying astronauts for their maiden spaceflight missions. The current NASA standard of a DXA *T*-score > −1.0 for the hip should remain the acceptable criterion for pre-flight skeletal health. However, because DXA technology cannot detect all of the effects of spaceflight on bones, the FE Strength Task Group recommends the inclusion of QCT-FE into the standard operating procedures for assessing astronaut skeletal health following prolonged space exposures. QCT-FE provides an index that is complementary to DXA aBMD and an understanding of how spaceflight affects hip integrity that DXA cannot. It was suggested by NASA personnel that, because younger persons are already at low risk for osteoporosis and fractures, even with aBMD *T*-scores < −2.5, it is not equitable to screen applicants to astronaut candidacy based exclusively upon a DXA test. QCT-FE modeling for hip bone load capacity would provide a secondary, science-based test to screen and authorize prioritized applicants, with *T*-scores < −1.0 but > −1.5, to progress through medical screening.

**Fig. 6:**
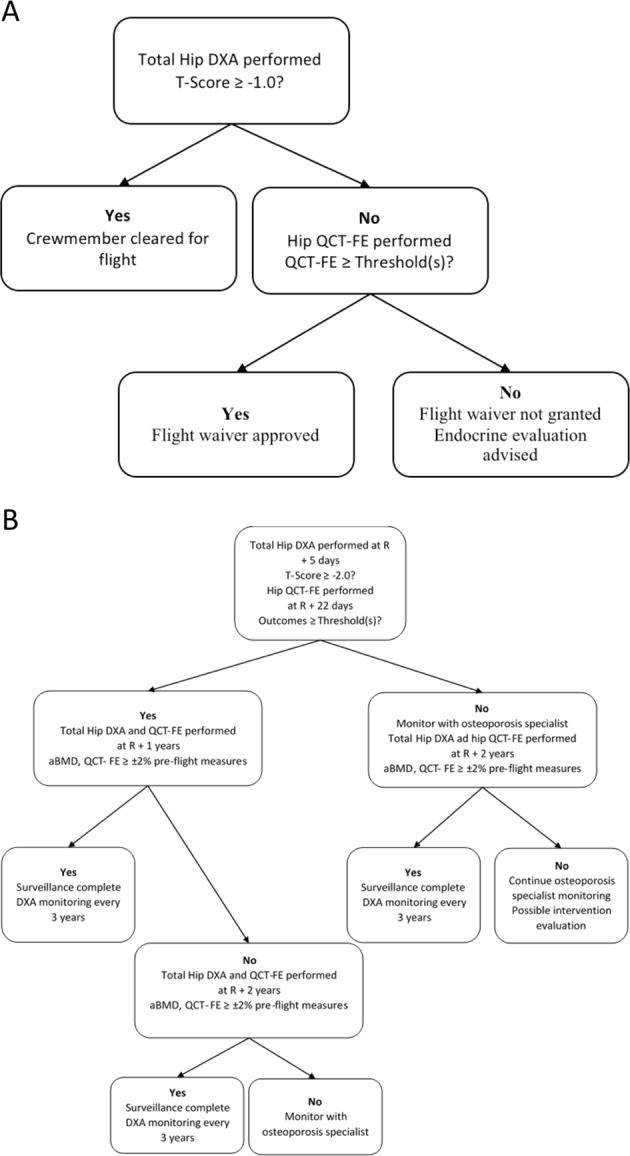
**a** Recommended standard operating procedure to integrate QCT-FE into current pre-flight monitoring standard operating procedure. QCT-FE is to be used for a biomechanical assessment of hip integrity. **b** Recommended standard operating procedure to integrate QCT-FE into current post-flight monitoring standard operating procedure. QCT-FE is to be used as a comprehensive index for monitoring post-flight skeletal health recovery

A proposed integration of QCT-FE into a standard operating procedure for a crewmember’s pre-flight skeletal assessment is presented in Fig. 6a and for post-flight assessment in Fig. 6b. The integration of QCT-FE would provide a more comprehensive assessment of skeletal health by providing additional indices, including estimated bone load capacity and vBMD for separate cortical and trabecular bone compartments that can change profoundly with spaceflight.

### Overall assessment

NASA is faced with the risks that the current clinical test (DXA) cannot fully detect skeletal changes with spaceflight, recovery, and may incompletely evaluate the efficacy of countermeasures. Due to these limitations, NASA may be inadequately assessing the actual fracture risk of individual astronauts. Post-flight risk management could be enhanced by pre- and post-flight QCT-FE modeling, as it provides a biomechanical index for bone health (i.e., the magnitude and orientation of mechanical loading that would cause the hip to fracture). Through the use of Fig. 4, QCT hip scans would be performed post-flight for long-duration astronauts to assess complete skeletal health recovery, within measurement error, which is not currently feasible by DXA alone. Although hip QCT scans would enable post-flight monitoring of full recovery, the FE analysis of QCT data also generates estimates of hip load capacity that could be used to inform engineering requirements for spacecraft or spacesuit designs, or to suggest modifying physical activity levels, to reduce likelihood of overloading the hip. As more longitudinal spaceflight data are collected and the modeling of applied hip loads enhanced, these recommendations should be further refined for specific mission scenarios.

## Methods

### Population DXA and QCT scanning

DXA and QCT scans were acquired from two separate population datasets for this study: the AGES cohort^47^ and the Rochester Epidemiology Project.^27,48^ Characteristics of both population cohorts can be referenced in Table 1. Data from the AGES cohort was previously analyzed and reported upon^21,29^ before inclusion in this study. From the AGES population (*N* = 139), a cohort consisting of male subjects with hip fracture were identified and approximately two age-matched non-fractured cases were selected from the pool of subjects. All participants provided written informed consent and the study was approved by the respective institution review boards (i.e., National Bioethics Committee in Iceland and the Institutional Review Board of the Intramural Research Program of the National Institute on Aging).

For the AGES cohort, QCT scans of the hip were acquired using a four-channel system (Sensation 4, Siemens Medical Systems, Erlangen, Germany) as previously described.^21,29^ A standardized helical scanning protocol (120 kVp, 140 mAs, 1 mm image thickness, Pitch = 1, and 4 mm collimation) was used for all subjects, extending from ~1 cm superior to the acetabulum to 5 mm distal to the lesser trochanter of the left hip. Within the scan field-of-view for all subjects, a density calibration phantom (Image Analysis, Columbia, KY, USA) was included to allow for Hounsfield Unit (HU) conversion to equivalent density values. No DXA data were available from the AGES cohort.

The cohort from the Rochester Epidemiology Project,^27,48^ referred to as the Mayo cohort, was determined from an age-stratified, random sample of Rochester, MN, USA, and spans ages 27–90 years for a total of 397 participants. Fracture data in the Mayo cohort were not analyzed in the present study, because few hip fractures occurred and because the fracture definition was not appropriate for our study (e.g., low trauma fractures were not distinguished from high trauma fractures).

Mayo DXA scans were performed using a single Lunar Prodigy System (GE Healthcare, Madison, WI, USA) scanner following a standardized scanning and analysis protocol, as previously reported.^49^ Hip QCT scans were performed using a four-channel CT scanner (LightSpeed Qx/I, GE Medical Systems, Waukesha, WI). A standardized imaging protocol (120 kVp, 80 mA, 0.8 s rotation time, 0.75 pitch, 20 s scan time, 2.5 mm image thickness, 7.5 mm/rotation table speed, and 10 mm collimation) was followed for all subjects.^27^ A density calibration phantom consisting of five tubes containing various concentrations of K_2_HPO_4_ in water (Mindways Model 3 Solid Calibration Phantom, Mindways Software, Inc., Austin, TX, USA) was included in the scan field-of-view for each subject for voxel-specific HU conversion to K_2_HPO_4_ equivalent density.

### Astronaut DXA and QCT scanning

Written informed consent was acquired from all astronauts whose data are reported in this paper per a protocol approved by the Institutional Review Board of NASA Johnson Space Center (Pro1531). As part of standard operating procedures,^11^ all eight crewmembers received pre- and post-flight DXA scans of the hip using a Hologic Discovery whole body densitometer (Hologic, Inc., Bedford, MA, USA). Scans were acquired on average 95 ± 34 days before flight and 8 ± 2 days after returning from flight. Both the left and right hips were scanned; however, only left hip data are reported here, as the left hip was used in the analysis across all components of this study. All DXA scans for an individual crewmember were acquired on the same scanner and analyzed by the same operator to ensure consistency. Scan acquisition procedures were performed as recommended by the manufacturer; however, modifications to the manufacturer’s analysis procedures were made, as previously described for reported spaceflight and bedrest data.^50^ Precision of the manual procedure is equivalent to that of the automated procedure available using current DXA analysis software.

Crewmember QCT scans were obtained at a local hospital (Houston Methodist St. John Hospital, Houston, TX, USA) using a single 64-channel CT scanner (Lightspeed VCT, GE Medical Systems, Waukesha, WI, USA). Astronauts provided informed consent in accordance with Institutional Review Boards of the collaborating institutions (NASA Johnson Space Center, University of California at San Francisco) for the collection of QCT measurements. QCT scans were collected following a standardized imaging protocol (140 kVp, 50 mA, 0.4 s rotation time, 0.984 pitch, 2.5 image thickness, and 40 mm collimation). A density calibration phantom, consisting of rods with concentrations of 0, 75, and 150 mg/cc calcium hydroxyapatite (Image Analysis, Columbia, KY, USA), was included in the field-of view for each scan to allow for voxel-specific HU conversion to calcium hydroxyapatite equivalent density.

Crewmembers consented to have FE estimates of hip bone load capacity, generated from their individual QCT scans, presented anonymously to the FE Strength Task Group II. QCT scans were performed for eight astronaut crewmembers who had also received DXA scans. Two QCT hip scans were performed for each astronaut, one prior to launch at 82 ± 26 days and the second upon return from flight at 10 ± 2 days. Six of the eight astronaut crewmembers were involved with previous spaceflight missions of varying durations (one long-duration spaceflight and five short-duration spaceflights); however, due to privacy concerns, specific details on previous length of spaceflight are not reported here, although should be further investigated. All astronauts performed a standardized exercise plan on the ARED during spaceflight, but excluded astronauts testing in-flight use of a bisphosphonate pharmaceutical countermeasure. Average time of flight for astronauts was 154 ± 19 days. The data analyzed and presented here are all from astronauts who are employed by NASA; no data from cosmonauts or astronauts employed by the international space agencies are reported in this manuscript.

### DXA and QCT scanner cross-calibration

Multiple DXA scanners were used in the analysis of data presented here. The Mayo cohort scans were performed using a Lunar Prodigy System, while the astronaut scans were performed on a Hologic Discovery. In order to compare the results of the two scanners, each scan was calibrated to the Hologic BMD values using previously defined linear equations.^51,52^

To ensure consistent measurements and to account for the use of multiple CT scanners, all QCT scans included in the analysis were cross-calibrated using an anthropometric hip phantom as previously described.^53^ In short, a custom phantom, simulating the pelvic area of the human body, was scanned on each independent CT scanner along with the calibration phantom that was used when scanning subjects at that site. Equivalent K_2_HPO_4_ or calcium hydroxyapatite densities derived from each scan measurement were related to actual density measurements of the anthropometric calibration phantom. These relationships were then used to determine a cross-calibration relationship between the calibrated equivalent density measurement on each of the scanners and the calibrated equivalent calcium hydroxyapatite density on the scanner for the AGES study. Each correction was then applied at the voxel level to obtain adjusted equivalent density values.

### QCT-FE analysis

FE analysis was performed for all subjects using standardized modeling techniques in NLS and NLF loading configurations. The NLS loading configuration has been shown to be highly reliable in assessing hip load capacity and in predicting fracture location,^39,54^ whereas NLF configuration simulates a conditional posterolateral fall on the hip. Both loading configurations have previously been thoroughly described and validated elsewhere.^10,39,55–57^ Briefly, subject-specific geometry and isotropic heterogeneous material properties were derived from the calibrated and cross-calibrated QCT density data. Each element of the model had nonlinear material properties defined by a density-dependent stress–strain curve consisting of a linear elastic region to a yield point, a perfectly plastic region, a strain softening region, and a second perfectly plastic region.^39^ Distortion energy failure theory was used as the yield criterion for model elements. To determine load capacity, a displacement was incrementally increased at the femoral head, whereas the distal femur was fully constrained, until the reaction force at the femoral head began to decrease. The load capacity was defined as the maximum reaction force.^29,39^ Least significant change in load capacity outcome measures, as previously reported, for NLS is 3.6% and for NLF is 11.3% (unpublished data).

### Statistical analysis

Statistical analyses were performed using R (v3.2.3, The R Foundation for Statistical Computing, Vienna, Austria). Linear regression was used to determine the coefficient of determination (*R*^2^) between parameters. For significance testing, the criterion *α*-level was set to 0.05. Correlation *p*-values were determined by testing the correlation coefficient to be different than zero (*H*_0_ = 0). All absolute and relative changes are presented as mean ± SD and range (minimum–maximum).

### Software availability

All software used in this study has previously been reported, as cited in this manuscript.

### Reporting summary

Further information on experimental design is available in the Nature Research Reporting Summary linked to this article.

## Supplementary information

Reporting Summary

## Data Availability

The QCT scan data that support the findings of this study are available from principal investigators Dr Shreyasee Amin (Rochester Bone Health Study) and Dr Vilmundur Gudnason (The Age, Gene/Environment Susceptibility Reykjavik Study) but restrictions apply to the availability of these data, which were made available for the current study and so are not publicly available. Data may be available upon reasonable request and with permission from the Drs Amin and Gudnason. Finite element models and associated finite element data may be made available upon reasonable request from Dr Lang. The datasets generated during the current study however are publicly available from the corresponding author on reasonable request.

## Supplementary information

**Supplementary information** accompanies the paper on the *npj Microgravity* website (10.1038/s41526-019-0066-3).
